# Mirror meetings with frail older people and multidisciplinary primary care teams: Process and impact analysis

**DOI:** 10.1111/hex.12905

**Published:** 2019-05-23

**Authors:** Sietske Grol, Gerard Molleman, Henk Schers

**Affiliations:** ^1^ Department of Primary and Community Care Radboud University Medical Center Nijmegen The Netherlands; ^2^ Department of Healthy Living Community Health Service Gelderland‐Zuid Nijmegen The Netherlands

**Keywords:** frail elderly, mirror meetings, multidisciplinary teams, patient perspective, process analysis, qualitative research

## Abstract

**Objectives:**

To analyse the process and impact of confronting multidisciplinary teams (MTs) in primary care with the experiences of frail older patients through mirror meetings (MMs), with the aim of supporting teams to organize care in a more patient‐oriented way.

**Methods:**

Process and impact analyses were performed using a mixed‐method approach. MMs were held with 14 frail older patients and four MTs comprising 23 health‐care professionals (HCPs) in primary care in the Netherlands.

**Results:**

Mirror meetings were feasible for frail older people living at home, although their recruitment was time‐consuming. Interaction between the patients was scarce, but they valued the opportunity to share their stories. HCPs preferred MMs overwritten reports about patient experiences. An impact analysis revealed four dominant professional areas for improvement: improve alignment with patient goals, improved communication with patients both orally and in writing, developing new pathways to connect with informal caregivers and an increased understanding that most HCPs are relative strangers to their patients.

**Conclusions:**

Mirror meetings are a relatively simple and promising method for exploring the ways in which frail older patients experience care.

**Practice implications:**

Given the right conditions, MMs could result in valuable processes to enable MTs to improve their working methods.

## 1 INTRODUCTION

A growing number of frail older people live in the community.^1^ Frailty is defined as the accumulation of functional deficits and diminishing physiological reserves.^2^ The complex health‐care needs of this group mean they can require a diverse range of primary health‐care professionals (HCPs) working together in an integrated patient‐centred way to meet their needs and life goals.^3^, ^4^, ^5^ Interprofessional collaboration, preferable by both care and welfare professionals, seems to be crucial for delivering integrated care.^6^, ^7^

In 2015, the Organisation for Economic Co‐operation and Development (OECD) emphasized the importance of integrating services from a patient's point of view rather than from a health‐care provider's perspective.^8^ Despite the fact that patient participation is high on the public agenda, studies have shown that multidisciplinary networks and teams often operate from a professional perspective,^9^, ^10^ while patient perspectives are rarely anchored in the design and working method of multidisciplinary teams (MTs).^11^ Studies into interprofessional collaboration have largely focussed on the viewpoint of the professionals^12^, ^13^, ^14^, ^15^ or on more quantitative patient outcomes.^16^, ^17^, ^18^ Researchers are hesitant to ask frail older people to participate in such studies due to their vulnerable state,^19^ which means that little is known about the experiences of these patients regarding their multidisciplinary care teams.^20^, ^21^ In a previous study, we found that HCPs want to increase the consideration given to patient perspectives in the organization of their care, but are unsure how to realize this.^22^, ^23^, ^24^

Many studies have investigated the quality and patient centeredness of care through the collection of survey data^25^ or focus group interviews^26^ about patient experiences, while others have researched the experience of patients invited to attend team meetings.^27^ Besides some papers on so‐called ‘mirror meetings’ (MMs; available in Dutch), we are not aware of any other publications in which patients have shared live feedback on care experiences with professionals.^28^, ^29^, ^30^, ^31^, ^32^ The available literature suggests that learning from patients is increasingly important. The experiential expertise of patients seemed to offer a stimulating perspective on the provision of care, and the mirror meetings are an effective and powerful tool for generating learning points for health‐care professionals and organizations from the patient's perspective. The open face‐to‐face confrontation appeals more to the individual HCP than survey results and other forms of indirect feedback. In order to achieve structural improvements in the provision of care from these learning points, a good follow‐up process is considered necessary.

### 1.1 Aim of the study

The objective of this study was to explore the feasibility of holding MMs with frail older patients and MTs in a primary care setting. Moreover, we were interested in the added value of MMs for MTs, as perceived by the HCPs. We aimed to detect potential adaptations that could be made to improve this method and organize multidisciplinary care for frail older people in a more patient‐centred way. Issues that were addressed during the study were as follows: (1) the feasibility of holding MMs for frail older people; (2) the process of holding MMs involving older patients, HCPs and a moderator; and (3) the impact of MMs on the MTs.

## 2 METHODS

### 2.1 General methodology

**Figure 1:**
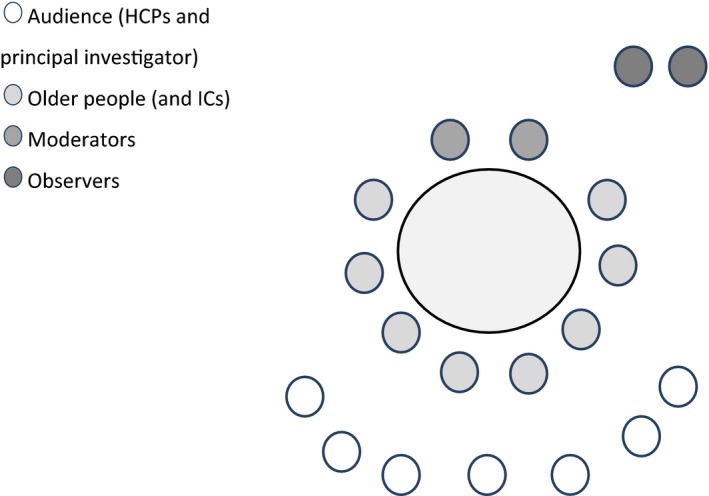
Arrangement of mirror meeting attendees

In order to present MTs with the perspectives of elderly patients living independently in the community, a MM methodology was used.^29^ The patients, seated in the ‘inner circle’, described their health‐care experiences while HCPs, seated in the ‘outer circle’, listened but were not allowed to speak (Box and Figure 1). The stories told by the older people were the focus of the meetings. MMs can be seen as a method of collecting narrative data.^33^

BOX 1 **What is a mirror meeting?**

1 A mirror meeting is a meeting of a group of patients, under the guidance of an independent moderator, in which the central question is how the patients experience the care they receive. The care providers involved are present only as listeners. The aim of the meeting is to increase the patient orientation of the care by making care providers aware of the patient perspectives. The success of a mirror meeting depends on the involvement of the care providers and requires them to be committed and have an open attitude to want to learn from patient feedback. The care providers are thus given a ‘mirror image’ by their own patients.^29^

### 2.2 Context of the study

This study was part of a project called PRECURO, which was focussed on gaining insights into the way in which multidisciplinary care (medical care, social services and community health services) in the Netherlands, in the region of Nijmegen, was organized for vulnerable older people living at home. Specific attention was paid to the experiences of older people, and the ways in which these experiences could be used to improve care. Data collection took place between February 2013 and June 2015, and consisted of interviews with frail older people, focus group meetings with HCPs, document analyses, observations of MT meetings, MMs and exchange meetings for MTs.

### 2.3 Recruitment of participants

In the Netherlands, all patients are registered with a general practitioner (GP). GPs deal with more than 95% of all presented medical problems and arrange referrals to secondary care when needed. Dutch GP services provide a comprehensive and patient‐oriented approach with a high continuity of care. GPs also co‐ordinate care for frail older patients with complex care needs.^34^, ^35^ From our network in the Nijmegen area of the Netherlands, we recruited four GP practices. We have approached these GP practices individually. All four agreed to participate. During the selection of the practices, heterogeneity was sought using the following criteria: geographical location, population served (deprived, commuter, city, village), years of experience with multidisciplinary elderly care and the scale of the GP practice setting. All participating GPs, including their most important stakeholders, aimed to organize integrated care for frail older people. GPs and their stakeholders together formed the MTs in our study (Table 1).

**Table 1:** Features of multidisciplinary teams

	MT 1 (N = 11)	MT 2 (N = 9)	MT 3 (N = 13)	MT 4 (N = 11)
Caregivers attending two focus group sessions	1 GP 1 practice nurse 3 community nurses 2 physiotherapists 1 occupational therapist 2 welfare workers 1 dietician	1 GP 3 community nurses 2 NHPSs 1 occupational therapist 1 welfare worker 1 health broker	1 GP 2 practice nurses 3 community nurses 1 mental health nurse 2 physiotherapists 1 welfare worker 1 health broker	2 GPs 2 practices nurses 2 community nurses 2 NHPSs 2 welfare workers 1 centre manager
Service area of MT	4800 patients	2150 patients	7000 patients	38 000 patients
Demographic features	Village with 22 555 inhabitants; 15% = 65+ y	Deprived neighbourhood in a medium‐sized city with 168 292 inhabitants; 14% = 65+ y	Commuters; neighbourhood with old village centre in medium‐sized city with 168 292 inhabitants; 14% = 65+ y	Small town with 41 775 inhabitants; 15% = 65+ y
GP setting	General practice	General practice	Health centre	Health centre

Abbreviations: GP, general practitioner; NHPS, nursing home physician specialist.

Three months before the MMs, all members of the four MTs were sent an announcement by email, which included the date and time of the MM. A month before the MM, they each received a detailed invitation by email, specifying the date, time, location and programme of the MM, in addition to an explanation of the aims of the meeting. The information sheet sent to the patients was also attached for their information.

For the PRECURO study, a total of 44 frail older people attending the four selected GP practices were interviewed (2.2.) about various subjects concerning the organization of their care. Potential frail older participants were selected by the GP and/or the practice nurse.^36^ Purposive sampling^37^ was used in terms of sex, age, living situation, degree of fragility and care needs to ensure a representative sampling of older people. Patients had to have been discussed in the MT meeting to be included. Those with severe cognitive impairments were excluded from participating. After obtaining written consent, the potential patients were approached. During the interviews, each patient was asked whether they wanted to participate in a MM. If the answer was positive, they were sent further details about the MM. Transport to the meeting was arranged if necessary. Patients received an information sheet explaining the purpose of the meeting and confirming their appointment.

### 2.4 Mirror meetings and process analysis

The MMs were guided by a practical manual, developed based on both the ‘manual for mirror meetings’ by Mul et al^29^ and the experience of a local patient organization, two representatives of which participated in the preparation, moderation and reporting of MMs. These representatives had experience with holding MMs with patients and professionals in various health‐care settings. After each MM, the manual was assessed to determine whether it required any adjustments.

Each MM lasted 90 minutes. The MMs took place at a location in the community familiar to the patients. If desired by the patient, an informal caregiver (IC) could be present. All meetings were audio‐taped and transcribed verbatim. A transcription protocol was made. The names of the patients were replaced by consecutive numbers on the transcripts to ensure anonymity and confidentiality. All meetings were observed^38^ by three occupational therapy bachelor students and captured in field notes.^39^

A semi‐structured topic list (Appendix S1), developed by the research team (SG and HS), was used to support all four meetings. The content of the topic list was determined by the research questions, supplemented with themes that emerged in the interviews with the elderly patients (2.2.). Also, data were collected from focus group interviews with the four MTs, in which the HCPs discussed the organization of care and mutual cooperation.^40^ In addition to this input, the HCPs were invited to submit topics for the MMs. The topic list structured the meeting. The following themes were addressed as follows: co‐ordination of care, the role of ICs and their contact with HCPs, MT meetings, personal health, care needs, the pathways to organize care and communication with care providers. At the end of the MM, the patients and HCPs were asked to evaluate the session. All patients received a modest gift as a token of appreciation.

During the MMs, the older people and their ICs sat around the table, chaired by the moderator(s), who were representatives of a local patient organization (Figure 1). The observers were seated in the background, in a position which allowed them to see the expressions of both the older participants and the HCPs.

The HCPs typically sat behind their patients so they could listen to them but could not directly look them in the eye, because this may have discomforted the patients. Professionals were allowed to ask questions at the end of the meeting to clarify what had been raised by the patients; however, they were not allowed to defend themselves or to enter into discussions with the patients (Box ).

The process analysis was carried out using the observations recorded in field notes and recordings and the results of the evaluation by patients and HCPs at the end of the MM.

BOX 2 **Rules for the mirror meeting**

1

Try to be open about personal experiences;
Each patient tells his/her own story;
You can express both appreciation and criticism;
Respect each other's privacy;
HCPs listen, but do not comment;
Patients participate in the role of an experience expert and not as a patient;
Minutes and tape recordings are transcribed anonymously.

### 2.5 Measuring impact

The impact of the MMs on MTs and their individual members was investigated in various ways. The care and welfare providers were observed during the MMs. Immediately after the MMs, they completed an online questionnaire about their experiences during the meeting. Two months after the MMs, the HCPs received a report about the MMs, which they discussed in their next MT meeting. After the discussion of the report, the principal investigator (SG) spoke with each GP (in one case also with the community nurse) to learn the results of this discussion. This can be seen as a form of member checking.^41^ In doing so, a number of open questions were asked, such as ‘What did you like about the mirror meeting?’ and ‘What actions will be taken as a result of the mirror meeting?’. Six months after the MMs, the MT members received an online questionnaire to measure the impact of the MMs.

### 2.6 Data analysis

Qualitative data were analysed using Atlas‐ti version 7.1. Open coding and thematic analysis were applied.^42^ Open questions from the questionnaires and interviews were analysed independently by two members of the research team. Transcripts were coded (open coding) and discussed together with the first and third author for clarification. Codes were grouped into categories (axial coding) and then into themes (selective coding). Themes and selected quotations were translated into English for this article.

The questionnaires were analysed using SPSS version 22. The transcription and analysis of the observation field notes were executed immediately after each MM so, if necessary, both the MM manual and the topic list could be adjusted for the next MM based on the qualitative observations.^33^, ^39^ The three student observers discussed their findings with the research team on a weekly basis.

## 3 RESULTS

### 3.1 Participants

Initially, we recruited 37 frail older people. After they were provided with more detailed information about the MMs by telephone, 14 decided not to participate. All of the reasons provided for this concerned the state of their health (eg feeling too depressed, having too much difficulty walking, being admitted to a care facility and cognitive impairments). After a second telephone call to the remaining 23 people 1 week before the meeting, another six people decided not to participate due to their poor health. At the last minute, three patients cancelled due to ill health. A total of 14 elderly people therefore participated in the MMs (Table 2).

**Table 2:** Characteristics of attendees of the mirror meetings

	MT 1	MT 2	MT 3	MT 4	Total
Frail older people					14
Gender					
Male	3	1	2	2	8
Female	1	3	1	1	6
Age					
65‐79	‐	2	‐	3	5
≥80	4	2	3	‐	9
Polypharmacy (≥5 medicines)	2	4	1	3	10
Amount of chronic diseases					
2‐4	1	2	‐	‐	3
5‐7	2	2	2	1	7
≥8	1	‐	1	2	4
General health, according to own view					
Excellent/very good/good	3	1	2	‐	6
Moderate/reasonable	‐	3	‐	3	6
Bad/very bad	1	‐	‐	‐	1
Missing	‐	‐	1	‐	1
Informal caregiver present^a^					
Family member	1	‐	4	‐	5
Neighbour/friend/acquaintance	1	1	‐	1	3
None	2	3	‐	2	7
Health‐care professionals					23
GP	1	1	1	2	5
Practice nurse	‐	2	1	2	5
Physiotherapist	‐	‐	‐	2	2
Occupational therapist	1	‐	‐	‐	1
Provider of social care service for older people	1	1	1	‐	3
Community nurse	1	1	2	1	5
Licensed vocational nurse	‐	‐	‐	1	1
Dietician	‐	‐	1	‐	1

a People could have more than one informal caregiver present.

Of the 14 patients, 57% were male, and the majority were over 80 years of age (64%), used multiple drugs (71%) and had ≥5 chronic disorders (79%). Half of the patients brought an IC to the meeting, either for logistical purposes or for mental support during the meeting. The moderator pointed out that the elder person herself/himself should be the primary speaker. Only factual information was sometimes provided by the IC.

All 31 HCPs who participated in the four MTs at the time the MM were organized were invited to take part, of whom 23 professionals (74%) participated. Two professionals were absent with notification, and six were absent without cancellation. In all meetings, at least one GP and one community nurse participated. In three of the four meetings, a practice nurse and/or a social care service provider for older people also participated. The other HCPs involved were physical and occupational therapists, a licensed vocational nurse and a dietician.

### 3.2 Process analysis

In all meetings, we observed that both patients and HCPs listened to each other attentively. After 30‐45 minutes, members of both groups showed physical agitation (eg not looking at the person who was speaking, looking at their phone, whispering to their neighbour). Some patients looked at the HCPs as if they were looking for endorsement or confirmation. With the exception of one MM, there was no interaction between the patients during the conversation. The moderator would ask a question to one patient, but the others did not react to nor reflect on the answer. All patients were absorbed by their own stories. Factual examples helped the conversation move forward and made the MM livelier.

As part of the process analysis, both patients and HCPs were invited to give feedback on the MM at the end of the session. The professionals were asked to share their observations and opinions, but discussions or explanations of earlier behaviour or actions were not allowed. Positive comments from the patients were that they felt it was an honest and inspiring meeting: ‘You could say what you wanted, whether it was positive or negative’. Patients found it pleasant to talk about their personal situation: ‘It made the support [of HCPs] tangible’. Both patients and HCPs made positive comments about the practical aspects of the MM: ‘nice venue’, ‘practical planning, at the end of the day’. Patients appreciated being able to look their HCPs in the face, where possible, and valued the opportunity to bring along a person they trusted. The caregivers stated that it was a pleasant and interesting conversation to listen to ‘You could really listen to how the patient experiences care’.

Both patients and HCPs commented negatively on the low number of patients participating in each MM, with the exception of one patient who stated ‘The low number of patients made me feel more comfortable to tell my story’. Two HCPs thought that some patients were not competent to participate due to psychiatric or cognitive impairments. One GP got very annoyed ‘I was especially [negatively] stimulated by the statements of one person [patient]. She told pertinent lies’. Some would have liked to be more involved with the recruitment of patients ‘I would have known a few suitable [patients]’. Some patients were difficult to understand, both due to a lack of microphones and the inadequate function or absence of hearing aids or dentures.

The MMs varied for the four MTs. After the first MM, three adjustments were made to the MM manual: the research team member who interviewed the patients in that particular community was seated at the table next to the moderator as a familiar face for the patients, ‘care’ was defined before starting the MM: ‘It's about your care: physiotherapy, general practice, home care, occupational therapy, daytime activities, mental healthcare, social services [names of local providers were given as an example]’, and the HCPs were seated to enable them to face the patients. A difference was observed between the first two and the last two MMs. In the first two MMs, the patients and HCPs seemed to be more distant from each other, while in the last two MMs, the patients and professionals clearly knew each other. These final two MMs took place in a primary care setting that served smaller communities (MT 1 and 2).

### 3.3 Impact analysis

The response rate to the questionnaire immediately after the MMs (T1) was 91% (n = 21). After 6 months (T2), it was 48% (n = 11). Between T1 and T2, four GPs and one community nurse were interviewed about the impact of the MM on their MT. To summarize the answers to the quantitative questions on T1, most HCPs (81%) preferred attending a MM over reading a report about the MM. Just over half of the HCPs stated that the MM highlighted surprising new points of view, and 62% found the MM inspiring. The purpose of the MM was clear to all HCPs, and the timing and duration were appropriate according to most HCPs. When analysing the results of the open questions from all questionnaires and interviews, five themes emerged (A) patients and ICs; (B) the topic list; (C) the moderator; (D) professional insights; and (E) the added value of the method.

### (A) Patients and ICs

Most HCPs found the number of patients participating in each MM too few. Furthermore, they would have preferred to play a larger role in the recruitment of patients for the MM. Also, they advocated for a more prominent role for the ICs during the MM, to supplement the information provided by the patients. Opinions differed regarding the participation of patients with cognitive or psychiatric problems. Some said ‘It was valuable that patients with psychiatric issues could also participate’, while others were of the opinion that ‘They do not know what is going on’. The generic opinion was that participation of this patient group should depend on the goal of the MM.

### (B) Topic list

Although the topics addressed during the MMs were recognizable for the patients, they did not fit seamlessly with the subjects on the minds of the HCPs. Several professionals suggested that the topic list should be composed by, or in collaboration with, the MT, based on their goals.

### (C) Moderator

The HCPs were not very enthusiastic about the moderator. They felt that the questions asked were too general: ‘Patients sometimes did not answer because they were presented with too much information’. Also, the moderator should have had more of an understanding of the local situation and the patients: ‘I did not find the moderator suitable because she had no connection with the interviewees’.

### (D) Professional insights

Health‐care professionals (HCPs) were surprised about the limited insight of the patients into their care: ‘Professionals are relative strangers to patients’. Another conclusion was that communication between the MT and their frail older patients should be improved and that the language used is often not understandable for this population: ‘.... align more with the perception of the patient’, ‘We should be focusing even more on the patient's goals and less on the points that we consider important’ and ‘Keep listening to the patient and respect what the care recipient wants’. Another conclusion made by the HCPs was that ‘The patient's family is really carer number one’. ICs should have a larger role in the care of this population, while bearing in mind that the patient's goals are the main focus: ‘Both formal and informal carers should stay close to the patient to find out what he or she wants’. Some HCPs found the target population too vulnerable for a MM: ‘Older people mostly consider only their own situation’.

### (E) Added value of the method

Different HCPs rated the added value of the MMs as being ‘no use all the way’ to ‘very valuable’. The perceived potential of the method was, among other things, its utility in the education of HCPs. One GP stated ‘The whole process worked as a kind of ‘peer‐to‐peer learning’, and ‘The MM can be used as an instrument in a quality improvement process. [It is] ideal for generating support. With a focus group you determine the theme of the improvement project, with the MM you identify areas for improvement that you can work on together’, Another GP stated ‘Mirror meetings can be very useful and fun. I would like to use the method more often in the future. It is an educational way to hear what patients think’. Some HCPs in one MT did not think that the MM would change anything about their working methods, citing the limited number of patients that participated and that the topics were not specific enough. They did consider the methodology to be promising, however, and would want to use it again. The majority of other HCPs did indeed find the method of MMs useful.

### 3.4 Insights and action

After the MMs and the discussion of the MM report in the MT meeting, the teams formulated the following points of action based on the experiences the patients shared with them:

To involve ICs in the care of their vulnerable relatives or friends, and find new ways to connect with them;
To visit the patient, prior to an MT meeting, to explain what the meeting is about and discuss the goals of the patient. After the MT meeting, the patient should then be informed about the outcomes of the meeting;
To provide better written information to the patient about ‘who is who’ (pictures of HCPs) and about the care plan.

## 4 DISCUSSION AND CONCLUSION

### 4.1 Discussion

#### 4.1.1 Summary

Mirror meetings with frail older people were found to be feasible. The patients valued being able to tell their story, and the MMs made the support of their HCPs more tangible. People with mild cognitive and psychiatric issues were able to participate, although not all HCPs considered their participation to be positive. In general, interactions between patients were scarce during the MMs. Both the patients and the HCPs were of the opinion that the minimum number of participating patients should be four. HCPs preferred attending a MM to reading a report about patient experiences. The topic list of the MMs did not always align with the goals of the MTs, and the HCPs felt that the moderator did not have a sufficient connection with the patients and their community. HCPs were surprised about the limited insight of the patients into their care and that HCPs are relative strangers to patients. The MMs resulted in the HCPs identifying specific points of improvement for their care, including paying more attention to ICs, placing more of an emphasis on the older person's goals and improving communication with patients and their ICs, both with regard to MT meetings and about the HCPs involved.

#### 4.1.2 Comparison with existing literature

As indicated in the introduction, little information was found on the subject of our study; however, we did find a book by Bijker,^43^ who argued that the important prerequisites for a successful MM are that the HCPs maintain an open mind and that the agenda is in line with the HCPs’ interests. These conditions improve the involvement of HCPs and increase the chance of the MM having an impact on care, a finding consistent with our own.

More studies have been performed on a number of related subjects. Lindberg et al^27^ evaluated the participation of frail older people in MT meetings within a hospital. The patients in their study also valued the opportunity to participate and share their views with HCPs.

Our experiences with the recruitment of patients were similar to those described in the existing literature, such as the challenges of travelling to the research site, the dropout rate throughout the study due to the deterioration of patient health, and the challenges of involving patients with cognitive and psychiatric conditions.^36^, ^44^

Many studies have elaborated on the importance of involving ICs for frail older people.^45^, ^46^, ^47^ Our study endorses these findings.

#### 4.1.3 Strengths and limitations

As far as we could establish, the MM methodology has not been written about in international literature before, despite being a relatively simple and elegant way to involve the voice of the patient in the process of organising care with MTs.

Methodological triangulation was used in both data collection (observations, audio‐taping and transcription, questionnaires, interviews) and the corresponding data analysis. Member checking was applied by sending the report of the MMs to the teams and reflect on the outcomes of the discussion of the report in the MT. This way of working makes qualitative research more robust.

The MM methodology used with this group was not piloted due to time pressures and limited resources. We did not perform further research into the experiences of the patients or the consequences for the care organization, although our material is suitable for this. If time had allowed us to do so, we would certainly have done this.

The experiment was performed with four teams in the Netherlands; however, we believe our results are also relevant for HCPs in other settings and countries because frail elderly people living independently and receiving multidisciplinary care are fairly universal: MTs throughout the Western World are striving for ways to hear the patient's voice, which could be achieved using MMs.

### 4.2 Conclusions

The results of our investigation demonstrate that MMs can play a role in clarifying patient experiences and their perceptions of care and MT collaboration for HCPs. Given the right conditions, MMs are an accessible method for all HCPs to become acquainted with patient views and experiences. A team looking to improve their working methods could recruit patients from their community, involve a moderator with knowledge of the community and the patient group and set up a MM. A manual and an independent party to facilitate the organization of the MM would be helpful. Our explorative study offers support for collaborative professionals in primary elderly care, who share an ambition of further anchoring the perspectives of their target population in the organization of their care.

### 4.3 Practice implications

With this research, we aimed to deliver usable information for HCPs that might help to bridge the gap between what older people need and what professionals deliver. The MM manual we developed proved useful and will be adjusted based on our study to improve its utility for MTs in primary care in the future. See Box for further practical issues concerning MMs.

BOX 3 **Additional findings: Practical issues of mirror meetings**

1

The maximum duration of a MM for both patients and HCPs is 45 minutes.
The minimum distance between the inner and outer circle is two metres.
Set a quiet environment without many distractions and ensure a smooth and organized approach. Provide enough food and beverages, a pleasant temperature, a warm welcome, etc.
Be aware that you are dealing with frail patients. They may cancel at any time, even on the day of the meeting.
Pay attention to audibility, including hearing aids and dental prostheses, and consider microphones.
The moderator should be someone who knows (of) the community, uses factual examples, probably meets the patients beforehand, but is independent of the (organization of the) professionals involved.
Some patients prefer to see the faces of their HCPs. Make an informed choice about where to position patients and carers.

For the further development of MMs, their effectiveness could be measured on a longitudinal basis by measuring patient experiences in response to changes in health‐care needs and the organization of care. The feedback to the MTs could also be monitored more effectively. MMs could be developed in collaboration with, and commissioned by, an MT, with the explicit goal of improving some parts of their care delivery, which would make MMs a strong component of the process of change and quality improvement for community‐based primary care. We would advise to investigate the impact of MMs on the patient by means of a questionnaire or interview. We would also recommend a process in which teams decide on quality improvement actions that they want to implement as a result of the MM. This process should be monitored in a quality cycle. A manual for MMs could help these teams to get started.

## AUTHORS’ CONTRIBUTIONS

SG contributed to the conception and design of the study, contributed to acquire funding for the study, collected, analysed and interpreted data, and drafted the manuscript. GM contributed to the design of the study, supported data interpretation, critically revised the manuscript and gave final approval of the version to be submitted. HS conceived and designed the study, acquired the funding for the study, supported data interpretation, critically revised the manuscript and gave final approval of the version to be submitted.

## CONSENT FOR PUBLICATION

All patients gave written consent for the use of anonymized quotations from the mirror meetings in presentations and publications arising from the research.

## INFORMED CONSENT AND PATIENT DETAILS

I confirm all patient/personal identifiers have been removed or disguised so the patient/person(s) described are not identifiable and cannot be identified through the details of the story.

## ETHICS APPROVAL AND CONSENT TO PARTICIPATE

None required, according to the Arnhem and Nijmegen Research Ethics Committee (file number 2017‐3518). According to the Committee, the respondents in our research were not subjected to any actions or behaviour that indicated that the research should be considered as a research under the Medical Research Act (WMO [Wet Medisch Onderzoek]). Therefore, it was not necessary for the CMO region Arnhem–Nijmegen or another recognized review committee to make a positive assessment. The participants signed an informed consent form to be included in this study.

## DATA AVAILABILITY

Data may be available on reasonable request from the principal investigator (SG). Mirror meeting data are not publicly available as they contain information that could compromise research participant's privacy and consent. The mirror meeting manual is momentarily only available in Dutch. On request from the principal investigator, an English version can be provided.
