# Supplementary material for: Mirror meetings with frail older people and multidisciplinary primary care teams: Process and impact analysis
Source: Health Expect. 2019 May 23;22(5):993–1002. doi: 10.1111/hex.12905 (PMC6803397; doi:10.1111/hex.12905)
Supplement: Supplementary file 1 [file HEX-22-993-s001.docx]

**Appendix A. Topic list guide for mirror meetings**

**Welcome and short introductions**

- The inner circle (‘patients’): name, age, living situation
- The outer circle: HCPs, observers, researchers.

**Preface**

“It’s about your care: physiotherapy, primary practice, home care, occupational therapy, daytime activities, mental health care, social services [*names of local providers are given as an example*].”

“It’s about the multidisciplinary team (MT): [*names of the local MT participants are provided*], district nurse(s), general practitioners, nursing home physician specialists (NHPS), elderly counsellors, occupational therapists, and people from home care talk about people like you every month in this district. They talk about your health, your living situation, your hobbies, whether you are lonely, whether you are content in your skin, whether you need a mobility scooter, etc.. There are so many subjects.”

“We would like to know what you think of the collaboration between the HCPs and the organisations from whom you receive care.”

**Conversation topics (sequence is variable)**

*Co-ordination of care:*

Who do you see as your first point of contact / co-ordinator / case manager / the person in charge of your care? What are your experiences with this central person in your care? Who can you contact if you have problems? Who do you ask for advice? What do you think would be the role of a central person in your care?

*Role of informal caregivers:*

Do you have an IC? With whom do they have contact, either with the care-providing people present or with other people who work in healthcare? How does that contact work? What is your opinion on the contact you have with your ICs? What is the role of your IC(s)? What do you think of that? Should it be different?

*MT meeting about care for the elderly:*

Do you know whether care providers sometimes discuss your care together? What are your experiences of this? What do you think will be discussed during these consultations [MT meetings]? What have you noticed about these consultations? What do you think about this? What feedback has been given to you or to your IC about the MT meeting? By whom?

The members of the MT [names of the members] would like to work with you to enhance your quality of life. They want to ensure that you can stay at home and as healthy as possible for as long as possible. They want you to think about that and talk to them about it.

What do you think of that? How could that be achieved? Have you already had a discussion about this with [name of the community nurse]; for example, about what you find important when it comes to:

- your health,
- your living situation,
- what you like to do (e.g., watching football, playing bridge, going to choir, attending daytime activities, attending church, walking, reading)
- your tools (mobility scooter, adjustments at home)?

They all want to know that. What do you think about them wanting to know more about you than just about "doctor things"? The general practitioner [name] explains it like this: the MT wants to build scaffolding around you and a safety net underneath. So that if you ‘fall’ you end up ‘well’. They prefer to do this together with you and your ICs. What do you think about this? What role would you like to have in this? Do you want to talk to the care providers yourself or does your IC do this for you? Can you sufficiently indicate your wishes and needs to your care providers?

*Patient need for help:*

For which activities do you receive help and who provides it (household, daytime activities, mobility scooter, medication)? Who arranged this help for you? What did you arrange yourself? Are you missing information about how to ask for help; for example, from home care, the GP or social services? What information do you need? Who could give this to you? Do you feel free to ask for the help of your neighbours, acquaintances, friends, family, or would you like to do this more often but do not know how? What could be of help to you?

*Communication / consultation / contact with your care providers*

What do you notice about the consultation between the care providers themselves (GP, physiotherapist, community nurse, domestic help)? Do they ask each other for advice when it comes to your care (e.g., daytime care, medical aids, medication, diabetes)?

What do you like about the current contact between you and your HCPs? Did you personally experience a situation in which you did not feel heard? What caused this feeling?

Have you experienced situations in which you did not understand what the care provider told you? Do you have tips for your care providers about their current way of communicating? If a doctor tells you something difficult, it may be nice if someone is there to support you (family, friend, neighbours). Do you agree with this? What do you think about your IC being present during a consultation with you and a care provider, for example the GP or someone from home care?

*Meetings and appointments*Do HCPs usually follow-up on what you discussed with them, e.g., if you talk to them about adjusting medication, requesting a mobility scooter, arranging day care, transfer from hospital to home? Can you mention points of improvement in this area?

*Co-ordination of care between care providers*

If for example a social worker does not know how to help you with a problem, do they call in another care provider? If for example you want to exercise more, would your GP call in the occupational therapist or physiotherapist? If you would like to go to church, but do not know how you could attend, can they help you with that?

Sometimes several care providers are involved because of one health problem (e.g., Parkinson's disease, back problems, mental health care). Do you notice that your care providers have contact with each other about the care they offer you?

**Evaluation**

To the patients:

- What did you think of the mirror meeting?
- What did you notice?
- What will stay with you?

To the HCPs:

- Inviting people to react. Interaction is allowed as long as HCPs don’t defend or explain themselves.
- Do you have any clarification questions?
- What did you think about the mirror meeting?

**Closing remarks**: Thank you all for your participation
